# Disturbance Modelling for Minimum Variance Control in Adaptive Optics Systems Using Wavefront Sensor Sampled-Data

**DOI:** 10.3390/s21093054

**Published:** 2021-04-27

**Authors:** María Coronel, Rodrigo Carvajal, Pedro Escárate, Juan C. Agüero

**Affiliations:** 1 Departamento Electrónica, Universidad Técnica Federico Santa María (UTFSM), Av. España 1680, Valparaíso 2390123, Chile; maria.coronelm@sansano.usm.cl (M.C.); rodrigo.carvajalg@usm.cl (R.C.); juan.aguero@usm.cl (J.C.A.); 2 Advanced Center for Electrical and Electronic Engiennering, AC3E, Av. Matta 222, Valparaíso 2580129, Chile; 3 Departamento de Ingeniería Electrica, Facultad de Ingeniería, Universidad de Los Andes, Av. Alberto Carnevali, Mérida 5101, Venezuela; 4 Instituto de Electricidad y Electrónica, Facultad de Ciencias de la Ingeniería, Universidad Austral de Chile (UACH), Genaral Lagos 2086, Valdivia 5111187, Chile

**Keywords:** adaptive optics, wavefront sensor, disturbances, modelling, identification, minimum variance controller, Whittle’s likelihood

## Abstract

Modern large telescopes are built based on the effectiveness of adaptive optics systems in mitigating the detrimental effects of wavefront distortions on astronomical images. In astronomical adaptive optics systems, the main sources of wavefront distortions are atmospheric turbulence and mechanical vibrations that are induced by the wind or the instrumentation systems, such as fans and cooling pumps. The mitigation of wavefront distortions is typically attained via a control law that is based on an adequate and accurate model. In this paper, we develop a modelling technique based on continuous-time damped-oscillators and on the Whittle’s likelihood method to estimate the parameters of disturbance models from wavefront sensor time-domain sampled-data. On the other hand, when the model is not accurate, the performance of the minimum variance controller is affected. We show that our modelling and identification techniques not only allow for more accurate estimates, but also for better minimum variance control performance. We illustrate the benefits of our proposal via numerical simulations.

## 1. Introduction

The last decades have marked the advent of the extremely large telescopes epoch in ground-based astronomy, in which astronomical observatories have experienced a growth in the aperture of their telescopes, see e.g., [1]. Currently, the Large Binocular Telescope (LBT) in Arizona, USA, reaches a combined effective aperture of 11.9 m, whilst the Gran Telescopio Canarias (GTC) in the Canary Islands, Spain, reaches an effective aperture of 10.4 m. This kind of modern telescopes is used for deep universe exploration as well as sharp high-definition images. However, the attainment of such images is subject to the inherent effects of the atmosphere and the vibrations of different equipment that are part of the telescope, see e.g., [2].

Adaptive optics (AO) is an optical technique that is used to improve astronomical images by compensating the effect of wavefront distortions caused by atmospheric turbulences and mechanical vibrations [3,4]. AO systems are very sensitive to vibrations acting in the propagation of the light [2,5,6]. Mechanical vibrations are typically induced by wind or elements within the instrumentation of the system, such as fans and cooling pumps. Hence, the modelling and mitigation of disturbances are a subject of increasing importance in many observatories [2,7,8,9,10]. This mitigation is achieved by deforming a deformable mirror (DM) in order to compensate the optical aberrations that are measured by a wavefront sensor (WFS), which, in turn, implies the need for both an accurate model of the complete AO system and adequate controllers.

In order to improve the quality of ground astronomical images, it is essential to obtain accurate models that define the dynamics of the plant that is comprised of the cascade connection of the DM and the WFS [11,12,13] and the accurate model parameters of all sources of noise and disturbances (turbulence and vibrations) that allow for implementing effective control techniques. For large telescopes (>8 m), disturbances become even more relevant, since the larger structures of this type of telescopes naturally oscillate according to different modes. In fact, disturbance mitigation is one of the key challenges for the next generation of extreme large telescopes [14,15].

In this paper, we focus on obtaining accurate disturbance models for the design of a minimum variance controller (MVC) in AO systems to improve the performance of the AO system. We present an extension of the work in [16,17,18,19] using continuous-time autoregressive (CAR) damped oscillators to model the disturbance and the Whittle’s likelihood technique to estimate the continuous-time parameters of the damped oscillators. We focus on the estimation of the continuous-time model parameters since the continuous-time model allows using different sampling intervals for the identification and the control scheme, which is necessary for more advanced control techniques, see e.g., [20]. We also analyse the impact of the disturbance model accuracy on the control performance, using a discrete-time transfer function of the disturbances for the design of the controller. From our analysis, we show that the control performance can drastically increase when the MVC is obtained from more accurate models. In our simulations, the MVC control performance improves in about 19% when the controller is designed using the model that was obtained from our proposed identification technique.

The structure of the paper is as follows: In Section 2, a general description of a typical AO system is presented. In Section 3, we present an equivalent AO system model and the sampled-data model for the disturbances in AO systems. In Section 4, the proposed sample-data model for the disturbances is presented. In Section 5, we show the disturbance identification using the non-linear least square (NLS) fitting method. In Section 5, we also introduce our proposed identification algorithm that is based on the Whittle’s likelihood method to estimate the parameters of the continuous-time disturbance model. In Section 6, we present the MVC design and evaluate the MVC performance subject to model error. Numerical results are presented in Section 7, where we show the benefits of our proposed model and identification algorithm in terms of the MVC performance. Finally, we present conclusions in Section 8.

## 2. AO Systems

**Figure 1:**
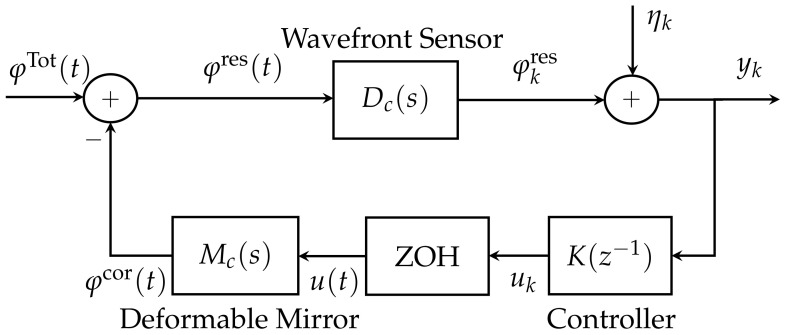
Block diagram for an AO closed-loop system.

Figure 1 shows the classical model of an AO system used in astronomy. This AO closed-loop system consists of a wavefront sensor, a deformable mirror $\left(M_{c}\left(s\right)\right)$, a controller $\left(K\left(z^{-1}\right)\right)$, and a zero order hold (ZOH) [6,21,22], where $s=\frac{d}{dt}$ should be understood as the derivative operator or the argument of the Laplace transform, and *z* is either the forward shift operator $\left(z\varphi_{k}=\varphi_{k+1}\right)$ or the argument of the Z-transform. The continuous-time signals $\varphi^{\mathrm{Tot}}\left(t\right)$, $\varphi^{\mathrm{cor}}\left(t\right)$ and $\varphi^{\mathrm{res}}\left(t\right)$ are the total disturbance (the amplitude of the disturbed wavefront; turbulence+vibrations), the correction disturbance (that corrects the wavefront), and the residual disturbance, respectively. The disturbed wavefront $\varphi^{\mathrm{Tot}}\left(t\right)$ is defined as the sum of the atmospheric turbulence effect $\varphi^{\mathrm{tur}}\left(t\right)$ and the different vibration sources effect $\varphi^{{\mathrm{vib}}_{l}}\left(t\right)$, l=1,…,m [5,6,22]. That is:

(1) $$\varphi^{\mathrm{Tot}}\left(t\right)=\varphi^{\mathrm{tur}}\left(t\right)+\varphi^{{\mathrm{vib}}_{1}}\left(t\right)+\varphi^{{\mathrm{vib}}_{2}}\left(t\right)+\cdots +\varphi^{{\mathrm{vib}}_{m}}\left(t\right),$$

where *m* is the number of vibration sources. The signal $\eta_{k}$ shown in Figure 1 is a discrete-time additive zero-mean white Gaussian noise with variance $\sigma_{\eta }^{2}$.

### 2.1. Wavefront Sensor

**Figure 2:**
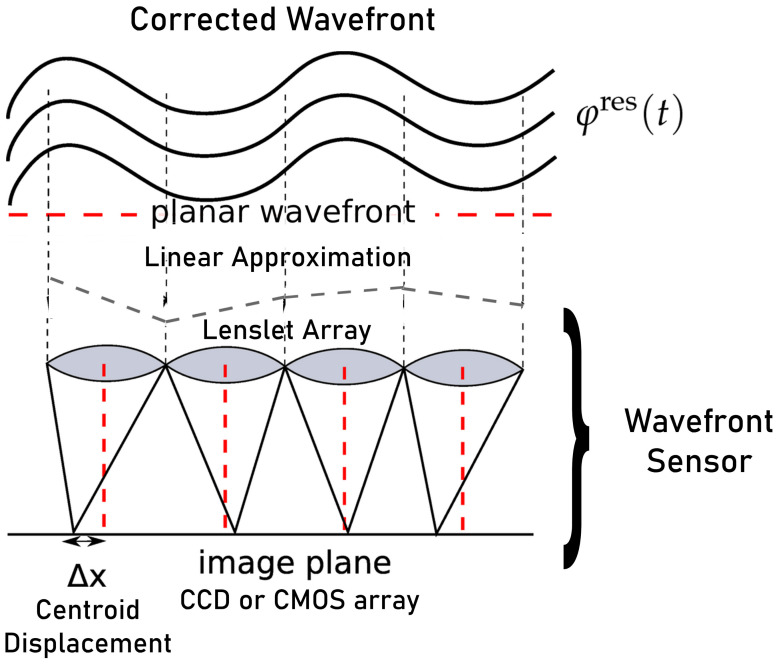
Wavefront sensor.

A wavefront sensor (Figure 2) is an optical device that is used to measure the aberrations of an image. It consists of an array of lenses (lenslets) focused on an CCD or CMOS array (image plane). The basic idea is to measure the displacement of the centroid with respect to the ideal wavefront (planar wavefront) centroid position. Afterwards, using this information we can obtain a reconstructed wavefront (a linear approximation). Because only tilts are measured, the Shack–Hartmann wavefront sensor is not capable of detecting discontinuous steps in the wavefront. The CCD o CMOS detector requires a period of time (integration time) to accumulate enough photons to sample the wavefront and obtain a centroid. This implies that the WFS integrates the incoming wavefront during the integration time.

### 2.2. Deformable Mirror

**Figure 3:**
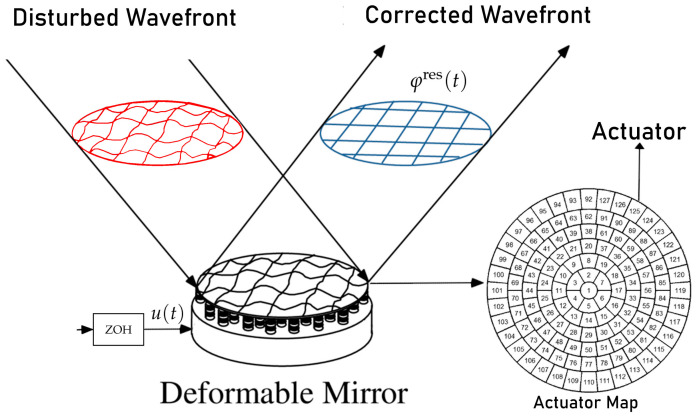
Operation of a deformable mirror.

A deformable mirror (DM) is an opto-mechanical reflective device that is capable of deforming the shape of its surface (see Figure 3). The shape of the DM can be controlled to correct the optical aberrations that are measured by a wavefront sensor in an AO system. This correction depends of the number of actuators, the actuator pitch (distance between actuator centres), the actuator stroke (maximum possible actuator displacement), the unfluence function i(characteristic surface shape corresponding to a single actuator), and the actuator coupling (displacement of the neighbours actuators).

### 2.3. AO Controller

The controller in an AO system sends the adequate signal to the deformable mirror to correct the disturbed wavefront that was measured by the WFS. Because the computation of the controller is demanding, the wavefront aberrations are represented by a linear combination of orthogonal basis elements. This linear combination allows for the separation of the control problem into independent modal controls, such as tip, tilt, and defocus [23,24].

The most used basis functions to describe aberrated wavefronts in optics are the Zernike polynomials, from which we obtain several orthogonal modes [23]. On the other hand, the varied nature of disturbances are perceived in different ways. For example, the mechanical vibration that is caused by excitations of on-site instruments usually exhibits narrow-band high-frequency components, the structural responses of large telescopes are within a low frequency band of natural resonances, and the atmospheric turbulences exhibit a (roughly) constant component below a corner frequency and a roll-off at high frequency on a Zernike basis [25]. In addition, the compensation of tip and tilt are one of the main tasks of AO [2,23,26]. Hence, an adequate identification of the disturbance source model is of great interest, which, in turn, allows for the development and implementation of effective control techniques.

A proportional-integral (PI) controller is a widely used control algorithm in AO [27,28,29]. However, with the construction of extremely large telescopes, the demanding requirements and challenging features increase, which causes the new generation of AO systems to require the implementation of more sophisticated identification and control techniques [1], such as linear-quadratic Gaussian (LQG) [1,5,7,29], minimum variance control (MVC) [26,30], or model predictive control (MPC) [31]. Notice that, in [26], the authors show that MVC is an equivalent representation of the typically used LQG controller for AO systems.

## 3. Disturbance Model in AO Systems

Typically, in the AO literature, the modelling and identification of disturbances in AO systems have been addressed using a second-order auto-regressive (AR(2)) discrete-time model with both time-domain data [26,29,32] and frequency-domain data [6,7,33,34,35]. In particular, in [35], an identification approach using a NLS fitting method was presented. This approach was successfully used to design a control strategy to mitigate the vibrations. Nevertheless, this model can present low accuracy at high frequencies (in the range $\left[4F_{s}/10,F_{s}/2\right]$, where $F_{s}$ is the sampling frequency). This, in turn, can lead to an unsatisfactory control performance in this high frequency range, especially for large telescopes. This behaviour is not desirable, since, for instance, high-order aberrations can be induced by misaligned components in an the AO system [23].

### 3.1. Equivalent AO System Model

In AO systems for astronomical observation, the WFS integrates the residual phase $\varphi^{\mathrm{res}}\left(t\right)$ during a time interval $\left[t_{k-1},t_{k}\right]$, where $t_{k}$ is a sampling time, accumulating photons over a time interval Δ [22]. Thus, the discrete-time residual phase is given by:

(2) $$\varphi_{k}^{\mathrm{res}}=\frac{1}{\Delta }\int_{t_{k-1}}^{t_{k}}\varphi^{\mathrm{res}}\left(t\right)dt;\;\;t_{k}=k\Delta ,$$

where $k\in N_{0}$ and Δ represent the sampling period. The corresponding discrete-time transfer function of the WFS in Equation (2) is given by [22]:

(3) $$D\left(z^{-1}\right)=D_{0}z^{-\mu },$$

where $D_{0}$ is the gain and μ is the measurement delay.

Typically, the transfer function of the DM is modelled as the following discrete-time transfer function [5,11,22,26,35]

(4) $$M\left(z^{-1}\right)=M_{0}z^{-\tau },$$

where $M_{0}$ is the gain and τ is the correction delay.

**Figure 4:**
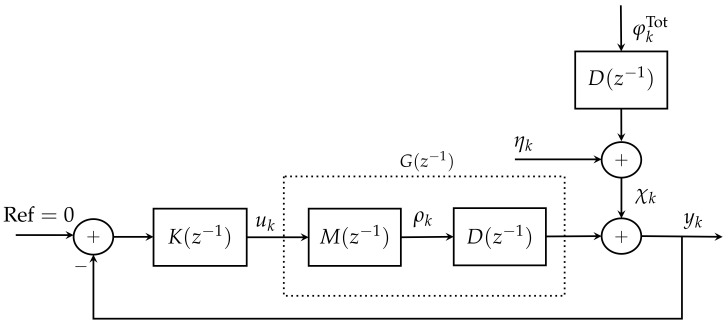
Equivalent block diagram model for AO system. The auxiliary variable $\rho_{k}=-\varphi_{k}^{\mathrm{cor}}$.

On the other hand, we consider that all of the elements in Figure 1 are linear. Subsequently, $\varphi_{k}^{\mathrm{res}}$ is a linear function of the past values of $\eta_{k}$. The output of the system presented in Figure 1 is given by

(5) $$\begin{array}{c} y_{k}=D_{0}\varphi_{k-\mu }^{\mathrm{res}}+\eta_{k}, \end{array}$$

where $\varphi_{k}^{\mathrm{res}}$ is the discrete-time residual phase that is given by:

(6) $$\varphi_{k}^{\mathrm{res}}=\varphi_{k}^{\mathrm{Tot}}-\varphi_{k}^{\mathrm{cor}},$$

and $\eta_{k}$ is a zero-mean white Gaussian noise. Subsequently, the variance of the output $y_{k}$ is given by:

(7) $$\mathrm{var}\left\{y_{k}\right\}=\sigma_{\eta }^{2}+D_{0}^{2}\mathrm{var}\left\{\varphi_{k-\mu }^{\mathrm{res}}\right\},$$

thus, minimising the variance of $y_{k}$ is the same as minimising the variance of $\varphi_{k}^{\mathrm{res}}$. Based on the equivalent block diagram for the AO system shown in Figure 1 that was presented in [26], a simple control system theory interpretation of this diagram is shown in Figure 4, where $K(z^{-1})$ is the discrete-time transfer function of the controller, $M(z^{-1})$ is the DM discrete-time transfer function, $D(z^{-1})$ is the WFS discrete-time transfer function, $u_{k}$ is the output of controller, and $y_{k}$ is the system output. Note that $G(z^{-1})$ is the equivalent discrete-time transfer function of the plant.

When considering the equivalent block diagram shown in Figure 4, the output signal is given by:

(8) $$y_{k}=-D_{0}\varphi_{k-\mu }^{\mathrm{cor}}+\chi_{k},$$

where

(9) $$\chi_{k}=\eta_{k}+D_{0}\varphi_{k-\mu }^{\mathrm{Tot}},$$

corresponds to the disturbances of the AO system. Subsequently,

(10) $$y_{k}=D_{0}\left[\varphi_{k-\mu }^{\mathrm{Tot}}-\varphi_{k-\mu }^{\mathrm{cor}}\right]+\eta_{k}.$$

Note that $\varphi_{k}^{\mathrm{Tot}}$ can be modelled in different ways. In Section 3.2 we present the typical model utilised in AO systems. In Section 4, we present our proposed model for the disturbances.

### 3.2. Classical Sampled-Data Model for Disturbances in AO Systems

Typically the continuous-time AO disturbance model is expressed, as follows [22]:

(11) $$\begin{array}{cc} \varphi^{\mathrm{Tot}}\left(t\right) & =\sum_{l=0}^{m}\frac{\beta_{l}}{s^{2}+4\zeta_{l}\pi \alpha_{l}s+{\left(2\pi \alpha_{l}\right)}^{2}}{\overset{\cdot }{\nu }}_{l}\left(t\right),\\ & =\sum_{l=0}^{m}\frac{\beta_{l}}{s^{2}+2\zeta_{l}\varpi_{l}s+\varpi_{l}^{2}}{\overset{\cdot }{\nu }}_{l}\left(t\right), \end{array}$$

where $\beta_{l}$ is the gain, $\alpha_{l}$ (Hz) is the natural frequency, $\zeta_{l}$ is the damping coefficient, and ${\overset{\cdot }{\nu }}_{l}\left(t\right)$ is a continuous-time zero-mean white Gaussian noise with variance $\sigma_{l}^{2}=1\delta_{D}\left(t\right),\forall l$ ($\delta_{D}\left(t\right)$ is the Dirac delta). We assume that the noises ${\overset{\cdot }{\nu }}_{l}\left(t\right),\;l=0,\cdots ,m$ are jointly uncorrelated. We use $\left(\varpi_{l}=2\pi \alpha_{l}\right)$ for simplicity in the presentation.

In AO, the disturbances in discrete-time are typically modelled as:

(12) $$\varphi_{k}^{\mathrm{Tot}}=\sum_{l=0}^{m}H_{l}\left(z^{-1}\right)\varepsilon_{l,k},$$

where $\varepsilon_{l,k}$ is a zero-mean white Gaussian noise with variance $\sigma_{\varepsilon_{l,k}}^{2}=1,\forall l$, jointly independent, $H_{0}\left(z^{-1}\right)$ is the discrete-time transfer function that is associated with the turbulence term and $H_{l}\left(z^{-1}\right)$, l=1,⋯,m, and the transfer function associated with the vibrations terms. The discrete-time transfer function $H_{l}\left(z^{-1}\right)$ is modelled using the following approximated auto-regressive (AR(2)) discrete-time representation [5,6,7,11,26,34,35]:

(13) $$H_{l}\left(z^{-1}\right)=\frac{\gamma_{l}}{1-a_{1_{l}}z^{-1}+a_{2_{l}}z^{-2}},$$

where $\gamma_{l}$, $a_{1_{l}}$ and $a_{2_{l}}$ are the parameters for the autoregressive model. For the vibrations models, the parameters are defined by:

(14) $$a_{1_{l}}=2e^{-\zeta_{l}\varpi_{l}\Delta }\cos\left(\varpi_{l}\sqrt{1-\zeta_{l}^{2}}\Delta \right),$$

(15) $$a_{2_{l}}=-e^{-2\zeta_{l}\varpi_{l}\Delta }.$$

**Remark** **1.** *In the approximated model, the mathematical expression for the gains $\gamma_{l}$ in (13) are not provided in the AO literature. Thus, they have to be estimated from the experimental data.*

The atmospheric turbulence is typically assumed to be time-invariant and statistically stationary for a fixed period of time [36,37,38]. This condition is referred to as the frozen-law approximation. It is widely understood that the model of the turbulence is very complex and a very accurate model will lead to a high computational load for AO systems. It has been shown in [6,39] that an adequate relaxation of the model can be achieved by fitting models that are simple enough for understanding (modelling) the corresponding phase distortion and carrying out fast computations (of the controllers), such as AR(1). In [40], it was shown that the AR(2) models better describe second order statistics of the atmospheric turbulence. In addition, in [20,34], an AR(2) model for the turbulences mode was proposed with the following parameters:

(16) $$a_{1_{0}}=2e^{-\zeta_{0}\varpi_{0}T}\cos\left(\varpi_{0}\sqrt{1-\zeta_{0}^{2}}T\right),$$

(17) $$a_{2_{0}}=-e^{-2\zeta_{0}\varpi_{0}T},$$

(18) $$\varpi_{0}=0.6\pi \left(n\left(i\right)+1\right)\frac{V_{0}}{D},$$

where n(i) is the radial order of the Zernike mode *i*, T is the exposure time of the system, D is the aperture diameter, and $V_{0}$ is the wind constant velocity. Hence, in this paper, we model the atmospheric turbulence as a second order system.

**Remark** **2.** *In the case that the atmosphere can be modelled as a sum of second order systems, our identification technique could be used to adequately estimate such a model provided the numbers of second order systems is sufficiently large enough.*

## 4. Proposed Modelling for Disturbances

In this section, we present a methodology to obtain an exact sampled-data model of the continuous-time disturbance model in Equation (11). The model in Equation (11) can be represented in state-space form as a CAR system:

(19) $$\overset{\cdot }{x}\left(t\right)=A_{c}x\left(t\right)+\kappa_{c}\Psi_{c}\left(t\right),$$

(20) $$\varphi^{\mathrm{Tot}}\left(t\right)=\overset{\cdot }{b}\left(t\right)=C_{c}x\left(t\right),$$

where $A_{c}\in R^{2m\times 2m}$, $x\left(t\right)\in R^{2m\times 1}$, $\kappa_{c}\in R^{2m\times m}$, $\Psi_{c}\in R^{m\times 1}$, $C_{c}\in R^{1\times 2m}$, *m* is the number of damping oscillators used, and the noise variance is $Q_{c}=\kappa_{c}\kappa_{c}^{T}$. These continuous-time system matrices are given by:

(21) $$A_{c}=\left[\begin{array}{ccccc} 0 & 1 & 0 & \cdots & 0\\ -\varpi_{1}^{2} & -2\zeta_{1}\varpi_{1} & 0 & \cdots & 0\\ \vdots & \vdots & \ddots & \vdots & \vdots \\ 0 & 0 & \cdots & 0 & 1\\ 0 & 0 & \cdots & -\varpi_{m}^{2} & -2\zeta_{m}\varpi_{m} \end{array}\right],$$

(22) $$\kappa_{c}=\left[\begin{array}{ccccc} 0 & \cdots & \cdots & \cdots & 0\\ \beta_{1} & 0 & \cdots & \cdots & \vdots \\ 0 & 0 & \ddots & \ddots & \vdots \\ \vdots & \beta_{2} & \ddots & \ddots & \vdots \\ \vdots & 0 & \ddots & \ddots & \vdots \\ \vdots & \vdots & 0 & \ddots & \vdots \\ \vdots & \vdots & \vdots & \ddots & 0\\ 0 & 0 & 0 & 0 & \beta_{m} \end{array}\right],\;\;\Psi_{c}\left(t\right)=\left[\begin{array}{c} {\overset{\cdot }{\omega }}_{1}\left(t\right)\\ {\overset{\cdot }{\omega }}_{2}\left(t\right)\\ \vdots \\ {\overset{\cdot }{\omega }}_{m}\left(t\right) \end{array}\right],$$

(23) $$C_{c}=\left[\;1\;0\;|\;1\;0\;|\;\cdots \;|\;1\;0\;\right],$$

where the vertical lines that are shown in Equation (23) represent the grouping of duple elements, corresponding to each term of the sum in Equation (11).

Note that it is necessary to define the auxiliary variable $\overset{\cdot }{b}\left(t\right)$ to obtain the exact discrete-time model [41].

We consider that the WFS integrates the residual phase in order to obtain the sampled-data model for the continuous-time disturbance model in Equation (11):

(24) $$\varphi_{k}^{\mathrm{res}}=\frac{1}{\Delta }\int_{t_{k-1}}^{t_{k}}\varphi^{\mathrm{res}}\left(t\right)dt,$$

this could be understood as an averaging anti-aliasing filter (AAF) that is used before taking samples (see [17,42]). For this kind of sampling, we define a system with an extended state $X\left(t\right)={\left[x^{T}\left(t\right)\;\;b\left(t\right)\right]}^{T}$ [41]. Subsequently, the continuous-time state-space model is as follows:

(25) $$\overset{\cdot }{X}\left(t\right)=AX\left(t\right)+\left[\begin{array}{c} \kappa_{c}\\ 0 \end{array}\right]\Psi_{c}\left(t\right),$$

where

(26) $$A=\left[\begin{array}{cc} A_{c} & 0\\ C_{c} & 0 \end{array}\right].$$

Thus, the corresponding discrete-time state equation model is given by [41]:

(27) $$X_{k+1}=e^{A\Delta }X_{k}+n_{k+1},$$

if we consider that $A_{c}$ is invertible, we have the following result [43]:

(28) $$e^{A\Delta }=\left[\begin{array}{cc} e^{A_{c}\Delta } & 0\\ C_{c}A_{c}^{-1}\left(e^{A_{c}\Delta }-I\right) & I \end{array}\right],$$

where I is the identity matrix and $n_{k+1}$ is a correlated discrete-time zero-mean white Gaussian noise with variance *Q* [41,44],

(29) $$n_{k+1}=\int_{0}^{\Delta }e^{\begin{array}{c} \left[\begin{array}{cc} A_{c} & 0\\ C_{c} & 0 \end{array}\right]\xi \end{array}}\left[\begin{array}{c} \kappa_{c}\\ 0 \end{array}\right]\Psi_{k+1-\eta }d\xi .$$

The variance *Q* and the exponential matrix can be obtained using that presented in [45]:

(30) $$e^{\begin{array}{c} \left[\begin{array}{cc} -A & Q_{c}^{*}\\ 0 & A^{T} \end{array}\right]\Delta \end{array}}=\left[\begin{array}{cc} P_{11} & P_{12}\\ 0 & P_{22} \end{array}\right],$$

where:

(31) $$Q_{c}^{*}=\left[\begin{array}{c} \kappa_{c}\\ 0 \end{array}\right]{\left[\begin{array}{c} \kappa_{c}\\ 0 \end{array}\right]}^{T},$$

then,

(32) $$\begin{array}{cc} e^{A\Delta } & =P_{22}^{T}, \end{array}$$

(33) $$\begin{array}{cc} Q & =\int_{0}^{\Delta }e^{A\xi }Q_{c}^{*}e^{{A\xi }^{T}}d\xi =P_{22}^{T}P_{12},\\ \; & =\left[\begin{array}{cc} Q_{11} & Q_{12}\\ Q_{12}^{T} & Q_{22} \end{array}\right], \end{array}$$

where $Q_{11}\in R^{2m\times 2m}$, $Q_{12}\in R^{1\times 2m}$ and $Q_{22}\in R^{1\times 1}$.

Thus, we have that

(34) $$\left[\begin{array}{c} x_{k+1}\\ b_{k+1} \end{array}\right]=\left[\begin{array}{cc} e^{A_{c}\Delta } & 0\\ C_{c}A_{c}^{-1}\left(e^{A_{c}\Delta }-I\right) & I \end{array}\right]\left[\begin{array}{c} x_{k}\\ b_{k} \end{array}\right]+n\left(t_{k+1}\right),$$

(35) $$\begin{array}{c} x_{k+1}=Ax_{k}+w_{k+1},\\ \varphi_{k+1}^{\mathrm{Tot}}\Delta =b_{k+1}-b_{k}=Cx_{k}+v_{k+1}, \end{array}$$

where:

(36) $$\begin{array}{cc} A & =e^{A_{c}\Delta }, \end{array}$$

(37) $$\begin{array}{cc} C & =C_{c}A_{c}^{-1}\left\{A-I\right\}, \end{array}$$

and the signals $w_{k+1}$ and $v_{k+1}$ are correlated discrete-time zero-mean white Gaussian noises with covariance matrix *Q*.

**Remark** **3.** *If we represent the plant $\left(G\left(z^{-1}\right)=M\left(z^{-1}\right)D\left(z^{-1}\right)\right)$ in state-space form as:*

(38) $$\begin{array}{cc} {\overset{\cdot }{x}}_{p}\left(t\right) & =A_{p_{c}}x\left(t\right)+B_{p_{c}}, \end{array}$$

(39) $$\begin{array}{cc} {\overset{\cdot }{b}}_{p}\left(t\right) & =C_{p_{c}}x_{p}\left(t\right), \end{array}$$

*where a ZOH and an averaging AAF are utilised, then the corresponding discrete-time system is given by [41]:*

(40) $$\begin{array}{cc} x_{p_{k+1}} & =A_{p}x_{p_{k}}+B_{p}u_{k}, \end{array}$$

(41) $$\begin{array}{cc} \frac{b_{p_{k+1}}-b_{p_{k}}}{\Delta } & =C_{p}x_{p_{k}}+D_{p}u_{k}, \end{array}$$

*where:*

(42) $$\begin{array}{cc} A_{p} & =e^{A_{p_{c}}\Delta }, \end{array}$$

(43) $$\begin{array}{cc} B_{p} & =\int_{0}^{\Delta }e^{A_{p_{c}}\xi }B_{p_{c}}d\xi , \end{array}$$

(44) $$\begin{array}{cc} C_{p} & =\frac{1}{\Delta }\int_{0}^{\Delta }e^{A_{p_{c}}\xi }d\xi , \end{array}$$

(45) $$\begin{array}{cc} D_{p} & =\frac{1}{\Delta }C_{p_{c}}\int_{0}^{\Delta }\int_{0}^{\xi }e^{A_{p_{c}}\phi }d\phi d\xi . \end{array}$$

On the other hand, the design of controllers, such as MVC, typically require only one discrete-time transfer function with one noise signal. In order to obtain this, we need to compute the spectral factorization $H(z^{-1})$ [46] via numerical approximation, solving a Riccati equation [46,47,48]. Subsequently, the proposed model of the disturbances is as follows (for more details on spectral factorization, see [46] (pp. 71–78)):

(46) $$\varphi_{k}^{\mathrm{Tot}}=H\left(z^{-1}\right)e_{k},$$

where $e_{k}$ is a discrete-time zero-mean white Gaussian noise with variance $\lambda^{2}$. Note that both $H(z^{-1})$ and $\lambda^{2}$ depend on the continuous-time parameters.

In order to develop an identification algorithm we define the discrete-time power spectral density (PSD) as follows [34,35]:

(47) $$S\left(e^{i\omega_{j}\Delta }\right)={\left|H(e^{i\omega_{j}\Delta })\right|}^{2}\lambda^{2},$$

where $i=\sqrt{-1}$, j=1,2,⋯,N and $0<\omega_{j}\leq 2\pi \left(F_{s}/2\right)$, and $\omega_{j}=\left(2\pi j\right)/\Delta$.

Finally, we summarize the procedure to obtain the discrete-time PSD shown in Algorithm 1.
**Algorithm 1** Discrete-time PSD1:Obtain the continuous-time state-space model of the disturbances using Equations (19) and (20)2:Obtain the continuous-time state-space model of the extended system in Equation (25)3:Compute the discrete-time state equation of the extended system in Equation (27)4:Compute the discrete-time system matrices, *A* and *C*, of the disturbances using Equations (36) and (37)5:Obtain the spectral factorization $H(z^{-1})$ and $\lambda^{2}$6:Obtain the discrete-time PSD in Equation (47)

## 5. Identification of Disturbances

In this section, we present two forms to obtain the disturbance model parameters. First, we show the method presented in [35], in which the typical AR(2) discrete-time disturbance model is used (see Equation (13)). Next, we propose to use the Whittle’s likelihood method to obtain the continuous-time disturbance parameters using the proposed model from the previous section.

### 5.1. Nonlinear Least Square Fitting Method

In [35], the authors estimated the disturbance frequencies, the damping coefficients, and the variances of the discrete-time noises (ε) signals assuming the model in Equation (12) and that the discrete gains were all equal to one. Using this method, in this paper, we assume the noise variance $\sigma_{\varepsilon }^{2}=1$ for all disturbance components, and we identify the disturbance frequencies, the damping coefficients, and the discrete gains. Despite these assumptions differing from the original approach in [35], it can be successfully implemented, since they are just a normalization procedure.

In order to formulate the NLS estimation algorithm, we define the vector of parameters to be estimated, ${\vec{\theta }}_{NLS}$, as follows:

(48) $${\vec{\theta }}_{NLS}={\left[\begin{array}{cc} {\vec{\zeta }}^{T} & {\vec{\gamma }}^{T} \end{array}\right]}^{T},$$

where $\vec{\zeta }={\left[\zeta_{1}\;\;\;\zeta_{2}\;\;\;\cdots \;\;\;\zeta_{m}\right]}^{T}$ is the vector that contains all of the damping coefficients, and $\vec{\gamma }={\left[\gamma_{1}\;\;\;\gamma_{2}\;\;\;\cdots \;\;\;\gamma_{m}\right]}^{T}$ is the vector that contains all of the discrete-time gains.

Following [35], we use the periodogram of the sampled-data and fit it with model ${\bar{S}}_{l}\left(e^{i\omega_{j}\Delta }\right)$ in Equation (49). The different frequencies (Hz) in $\vec{\alpha }={\left[\alpha_{1}\;\;\;\alpha_{2}\;\;\;\cdots \;\;\;\alpha_{m}\right]}^{T}$ were found in the periodogram as the frequency peaks.

The discrete-time PSD is given by:

(49) $${\bar{S}}_{l}\left(e^{i\omega_{j}\Delta }\right)={\left|\frac{\gamma_{l}}{1-a_{1_{l}}e^{-i\omega_{j}\Delta }-a_{2_{l}}e^{-2i\omega_{j}\Delta }}\right|}^{2},$$

and the NLS cost function $F_{l}\left(\theta_{NLS}\right)$ is obtained from

(50) $$\epsilon_{l}\left(e^{i\omega_{j}\Delta }\right)=10\log\left[\frac{I_{l}\left(e^{i\omega_{j}\Delta }\right)}{{\bar{S}}_{l}\left(e^{i\omega_{j}\Delta }\right)}\right],$$

where $I(e^{i\omega_{j}\Delta })$ is the periodogram of the sampled-data series $\varphi^{\mathrm{Tot}}$, and it is given by:

(51) $$I\left(e^{i\omega_{j}\Delta }\right)={\left|\frac{1}{\sqrt{N}}\sum_{t=1}^{N}\varphi_{t}^{\mathrm{Tot}}e^{-i\omega_{j}\Delta }\right|}^{2},$$

then,

(52) $$F_{l}\left({\vec{\theta }}_{NLS}\right)=\frac{1}{2}\epsilon_{l}{\left(e^{i\omega_{j}\Delta }\right)}^{T}\epsilon_{l}\left(e^{i\omega_{j}\Delta }\right).$$

Note that the periodogram must be divided in *m* sections according to the frequencies that were previously obtained, since each AR(2) system is fitted independently (not jointly) of the others.

Finally, the estimation problem can be written as the following optimisation problem:

(53) $${\hat{\theta }}_{NLS_{l}}=\arg\min_{{\vec{\theta }}_{NLS_{l}}}\;\left(F_{l}\left({\vec{\theta }}_{NLS}\right)\right),\;\;s.t.\;\;\left\{\begin{array}{c} 0<\zeta_{l}<1\\ \gamma_{l}>0 \end{array}\right.,\;l=0,\cdots ,m.$$

### 5.2. Whittle’s Likelihood

The Whittle’s likelihood function is a widely used frequency-domain approximation of the traditional likelihood function [49,50,51].

It is well known that the (time-domain) Whittle log-likelihood function is given by:

(54) $$\ell_{N}\left(\vec{\theta }\right)=-\frac{1}{N}\sum_{j=1}^{N}\log S\left(e^{i\omega_{j}\Delta }\right)-\frac{1}{N}\sum_{j=1}^{N}\frac{I(e^{i\omega_{j}\Delta })}{S(e^{i\omega_{j}\Delta })},$$

where *N* is the data length and $\vec{\theta }$ is the vector of parameters to be estimated that is given by:

(55) $$\vec{\theta }={\left[\begin{array}{ccc} {\vec{\alpha }}^{T} & {\vec{\zeta }}^{T} & {\vec{\beta }}^{T} \end{array}\right]}^{T},$$

$\vec{\alpha }={\left[\alpha_{1}\;\;\;\alpha_{2}\;\;\;\cdots \;\;\;\alpha_{m}\right]}^{T}$ is the vector that contain all the natural frequencies (in Hz), $\vec{\zeta }={\left[\zeta_{1}\;\;\;\zeta_{2}\;\;\;\cdots \;\;\;\zeta_{m}\right]}^{T}$ is the vector that contains all the damping coefficients, and $\vec{\beta }={\left[\beta_{1}\;\;\;\beta_{2}\;\;\;\cdots \;\;\;\beta_{m}\right]}^{T}$ is the vector that contains all the gains. In Equation (54) $S(e^{i\omega_{j}\Delta })$ is the discrete-time PSD of the disturbances given in Equation (47) and $I(e^{i\omega_{j}\Delta })$ is the periodogram that is given in Equation (51).

Notice that the Whittle log-likelihood is a function of the continuous-time system parameters (to be estimated) $\vec{\theta }$, since the spectrum $S(e^{i\omega_{j}\Delta })$ depends on them.

Afterwards, the ML estimation problem is given by:

(56) $${\hat{\theta }}_{\mathrm{ML}}=\arg\min_{\vec{\theta }}\;-\ell_{N}\left(\vec{\theta }\right),\;\;s.t.\;\;\left\{\begin{array}{c} 0<\vec{\alpha }<F_{s}/2\\ 0<\vec{\zeta }<1\\ \vec{\beta }>0 \end{array}\right..$$

Finally, we summarize our identification technique in Algorithm 2.
**Algorithm 2** Identification algorithm1:Compute the periodogram $I(e^{i\omega_{j}\Delta })$ using Equation (51) with $\omega_{j}=\left(2\pi j\right)/\Delta$, and j=1,2,⋯,N2:Choose an initial guess ${\hat{\theta }}^{\left(0\right)}$3:Obtain the discrete-time spectrum $S(e^{i\omega \Delta })$ in function of the vector of continuous-time parameters $\vec{\theta }$ using the Algorithm 14:Define the Whittle’s likelihood using Equation (54)5:Solve the ML estimation problem in Equation (56) e.g., using Matlab^®^ fmincon function

## 6. MVC Performance in AO Systems

### 6.1. Minimum Variance Control Design

In classical AO, the goal is to mitigate the wavefront distortions that are caused by atmospheric turbulences and mechanical vibrations in the astronomical images. That is equivalent to minimising the continuous-time residual phase variance, $\mathrm{var}\left\{\varphi^{\mathrm{res}}\left(t\right)\right\}$[22]. However, in [22], the authors demonstrated that minimising the continuous-time residual phase variance is equivalent to minimising the discrete-time residual phase variance, $\mathrm{var}\left\{\varphi_{k}^{\mathrm{res}}\right\}$.

The goal of MVC is to minimise the variance of the output signal $\mathrm{var}\left\{y_{k}\right\}$[42]. For simplicity of the presentation, we consider that the measurement noise, $\eta_{k}$, is an independent and identically distributed (iid) signal. We also assume that the atmospheric turbulence $\varphi^{\mathrm{tur}}$, the vibrations $\varphi^{\mathrm{vib}}$, and the measurement noise η, are mutually independent. In this setup, the measurement noise $\eta_{k}$ is assumed negligible. The atmospheric turbulence and vibrations will be modelled using the same model structure with different parameters. Thus the output of the AO loop is given by:

(57) $$y_{k}=z^{-d}D_{0}M_{0}u_{k}+H\left(z^{-1}\right)e_{k},$$

where d=μ+τ and $u_{k}$ is the output of the controller. $H(z^{-1})$ is the discrete-time transfer function obtained from the spectral factorization [46] of the spectrum of the signal $\chi_{k}$ in Equation (9). The signal $e_{k}$ is a zero-mean white noise with variance $\lambda^{2}$ that is also obtained from the spectral factorization procedure.

In order to obtain the MVC [42,52] we rewrite $H(z^{-1})$ as

(58) $$H\left(z^{-1}\right)=F\left(z^{-1}\right)+z^{-d}R\left(z^{-1}\right),$$

where $F(z^{-1})$ is a finite impulse response (FIR) filter that corresponds to the first *d* samples of the impulse response of $H(z^{-1})$, given by:

(59) $$F\left(z^{-1}\right)=1+f_{1}z^{-1}+f_{2}z^{-2}+\cdots +f_{d-1}z^{-(d-1)},$$

where $f_{\iota }$ is the ιth coefficient of the FIR filter $F(z^{-1})$, and $R(z^{-1})$ is a causal filter that can be obtained from Equation (58):

(60) $$R\left(z^{-1}\right)=\left(H\left(z^{-1}\right)-F\left(z^{-1}\right)\right)z^{d}.$$

Finally the discrete-time transfer function of the minimum variance controller is given by [42,52]:

(61) $$\begin{array}{c} K\left(z^{-1}\right)=\frac{R(z^{-1})}{F\left(z^{-1}\right)D_{0}M_{0}}, \end{array}$$

which corresponds to the controller in Figure 4.

### 6.2. Performance of MVC Subject to Model Error

It is well known that the performance of MVC is greatly affected by the accuracy of the model utilised in the design of the controller [42]. In order to analyse the effect of model error in its performance, we model the estimates of the disturbance transfer function in terms of the *true* value and an error term as:

(62) $$\begin{array}{c} \hat{H}\left(z^{-1}\right)=H\left(z^{-1}\right)\left(1+H_{E}\left(z^{-1}\right)\vartheta \right), \end{array}$$

where $H(z^{-1})$ is the *true* discrete-time transfer function and $H_{E}\left(z^{-1}\right)\vartheta$ is the multiplicative model error. The subscript E indicates multiplicative errors, whilst the parameter ϑ represents the gain of the multiplicative model error. When carrying out an identification procedure, the gain of the multiplicative model error can be set to ϑ=1 in order to obtain and subsequently analyse $H_{E}\left(z^{-1}\right)$. In some cases, a different set-up (e.g., a different measurement noise variance) could lead to the same $H_{E}\left(z^{-1}\right)$, but with a different gain.

In Table 1, we present the parameters of a continuous-time disturbance model that is comprised of 6 oscillators. To evaluate the performance of the MVC for this model, it is necessary to obtain the spectral factorization of $\chi_{k}$ by solving a Riccati equation [46,47,48].

**Figure 5:**
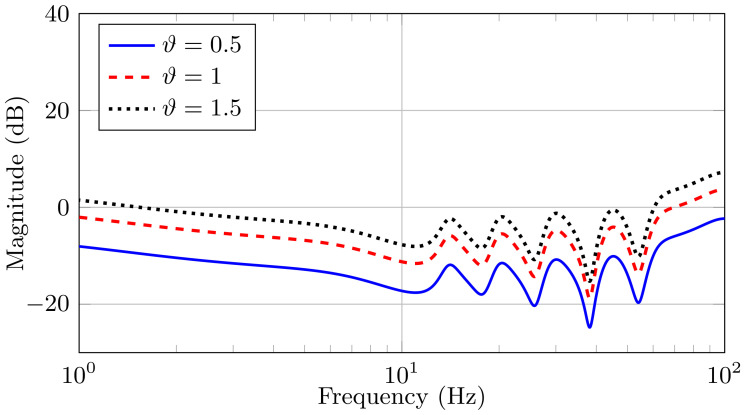
Frequency response of the multiplicative model error $H_{E}\left(z^{-1}\right)\vartheta$ for different values of ϑ.

In order to illustrate the effect of the error term on the estimated disturbance transfer function, we consider the continuous-time system (see Equation (11)) that is defined by the parameters in Table 1. Utilizing Algorithm 1, we obtain the corresponding PSD. We estimate the system model from the NLS method that is presented in [35] and, by fixing ϑ=1, we obtain the multiplicative model error $\left(H_{E}\left(z^{-1}\right)\right)$ using Equation (62). Finally, we select different values of ϑ and obtain the frequency response of the different multiplicative model errors. Figure 5 shows these frequency responses. We observe that there is uncertainty in the magnitude of the frequency peaks, since they correspond to the different natural frequencies of the oscillators in the model. We consider this uncertainty as multiplicative model error since the natural frequency of the disturbance $\left(\varpi_{l}\right)$ are “easy” to obtain (e.g., using the power spectral density), but the damping coefficients $\left(\zeta_{l}\right)$ and the gains $\left(\gamma_{l}\right)$ are not necessarily easy to obtain.

**Figure 6:**
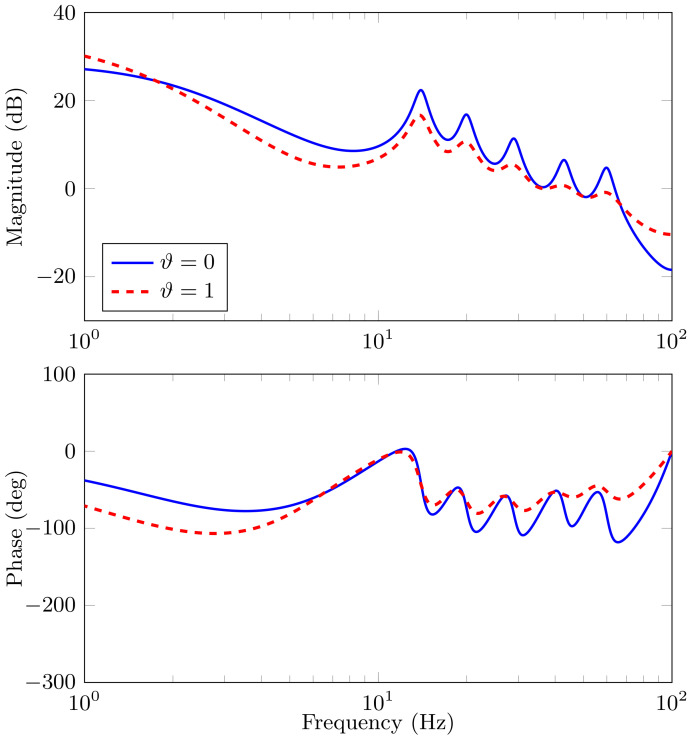
Frequency Response of the *true* discrete-time disturbance transfer function and the estimated discrete-time disturbance transfer function. The solid blue line represents the frequency response of the *true* disturbance model, $H(z^{-1})$, and the dashed red line represents the frequency response of the estimated model with ϑ=1.

For a more detailed analysis, in Figure 6 we present the *true* frequency response of the *true* disturbance discrete-time transfer function for this example and the estimated discrete-time transfer function using the method in [35] (with ϑ=1). The *true* system discrete-time transfer function was obtained from the continuous-time equation in Equation (11), the parameters in Table 1, and utilising Algorithm 1. We observe that, for some frequencies, the phase exhibits a very different behaviour when compared to the *true* frequency response, especially at low frequencies.

On the other hand, the performance of MVC can be obtained using the sensitivity function (S) [23,42,48]. The sensitivity function represents the closed-loop transfer function between the external perturbations and system output. The sensitivity function of the AO system in Figure 4 is defined as:

(63) $$S\left(z^{-1}\right)=\frac{1}{1+K\left(z^{-1}\right)G\left(z^{-1}\right)},$$

thus, when considering the disturbance model with uncertainty $\hat{H}\left(z^{-1}\right)=\hat{F}\left(z^{-1}\right)+z^{-d}\hat{R}\left(z^{-1}\right)$, we obtain

(64) $$S\left(z^{-1}\right)=\frac{\hat{F}\left(z^{-1}\right)}{\hat{F}\left(z^{-1}\right)+z^{-d}\hat{R}\left(z^{-1}\right)}=\frac{\hat{F}\left(z^{-1}\right)}{\hat{H}\left(z^{-1}\right)}.$$

If the *true* $H(z^{-1})$ was known $\left(\hat{H}\left(z^{-1}\right)=H\left(z^{-1}\right)\right)$, the output of the system can be obtained from the definition of the sensitivity as [48]:

(65) $$\begin{array}{c} {\hat{y}}_{k}=S\left(z^{-1}\right)H\left(z^{-1}\right)e_{k}=F\left(z^{-1}\right)e_{k}. \end{array}$$

From the last expression, the variance of the output signal is given by:

(66) $${\mathrm{var}}_{y_{0}}=\mathrm{var}\left\{y_{k}\right\}=\left(1+f_{1}^{2}+f_{2}^{2}+\cdots +f_{d-1}^{2}\right)\lambda^{2}.$$

However, since we only have an estimation of $H(z^{-1})$, defined as $\hat{H}\left(z^{-1}\right)$, the expression for the output signal is given by:

(67) $$\begin{array}{c} {\hat{y}}_{k}=S\left(z^{-1}\right)H\left(z^{-1}\right)e_{k}=\frac{\hat{F}\left(z^{-1}\right)H\left(z^{-1}\right)}{\hat{H}\left(z^{-1}\right)}e_{k}. \end{array}$$

Notice that the model mismatch between the *true* and estimated transfer functions has the effect of increasing the output signal variance, Therefore, the identification of the AO system is an important issue in the attainment of good control performance, which, in turn, allows for improving the quality of astronomical images.

### 6.3. Control Performance under Model Mismatch

In order to evaluate the impact of model accuracy on the control performance, we define the following coefficient as a performance metric:

(68) $$E=\left(\frac{{\mathrm{var}}_{\hat{y}}}{{\mathrm{var}}_{y_{0}}}-1\right)100\%,$$

where ${\mathrm{var}}_{\hat{y}}$ is the output variance using the estimated disturbance transfer function $\hat{H}\left(z^{-1}\right)$ and ${\mathrm{var}}_{y_{0}}$ is output variance obtain when the disturbance model matches the *true* disturbance, $\hat{H}\left(z^{-1}\right)=H\left(z^{-1}\right)$.

Finally, in order to illustrate the performance of MVC, we use a mirror gain $M_{0}=1$ and WFS gain $D_{0}=1$. We also select a measurement delay μ=1 and correction delay τ=1, i.e., d=2. Subsequently, using the *true* disturbance transfer function $\left(H\left(z^{-1}\right)\right)$ and the estimated disturbance transfer function $\hat{H}\left(z^{-1}\right)$ we can obtain the sensitivity function and the output variance to evaluate the performance of MVC. Table 2 shows the performance (*E*) for different values of ϑ, where it is clear that ϑ=0 is the ideal case, i.e., the estimated model without uncertainty. The E(%) denotes the percentage variation of the output variance with respect to the ideal case. We observe that the model accuracy has a big impact on the performance of MVC.

## 7. Numerical Example

**Figure 7:**
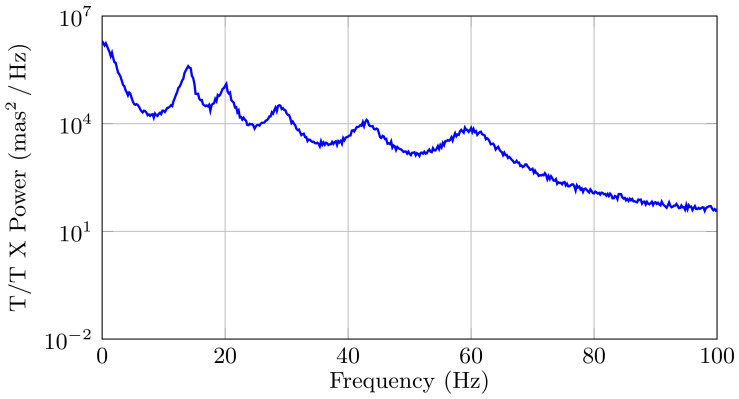
Discrete-time PSD $\left(|H(e^{i\omega_{j}\Delta }){|}^{2}\lambda^{2}\right)$ of the disturbance signal.

**Figure 8:**
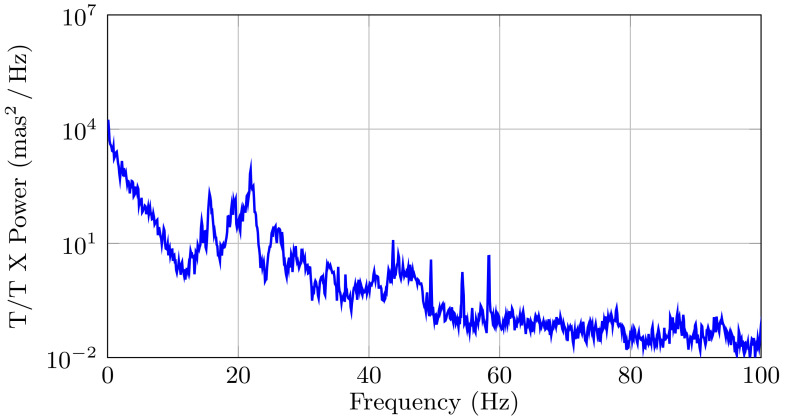
Discrete-time PSD of the turbulence-plus-vibrations perturbation signal in the Clay Telescope at the Las Campanas Observatory. 2014b observing run by the University of Arizona MagAO Team on the night of 31 October 2014.

In this example, we consider the six damped oscillators that are shown in Table 1 (which corresponds to the numerical example presented in [26]), and we consider that this is the *true* system. Figure 7 shows the discrete-time PSD of the disturbances, where we can clearly observe the disturbance frequencies. Note that this example has a similar shape and structure as the ones obtained during the 2014b observing run by the University of Arizona MagAO Team on the night of 31 October 2014, at the Clay Telescope in Las Campanas Observatory [26], which we include in Figure 8 for the completeness of the presentation. However, the PSD in Figure 8 corresponds to the data that were obtained in closed-loop, while the simulated data in Figure 7 are in an open-loop. Because of this fact, both of the PSDs have the same resonance peaks, but different magnitudes.

### 7.1. Disturbance Identification

We identify the continuous-time system parameters from a set of simulated data (generated with the six damped oscillators shown in Table 1) using the proposed system model in Equation (46) with the Whittle’s likelihood identification technique, and the system model in Equation (13) with the NLS fitting method proposed in [35]. We utilise a sampling period of Δ=5 ms, 100 Monte-Carlo (MC) simulations, and two data lengths, namely N=500 and N=1000. The estimation problems in Equations (53) and (56) are solved with a local optimisation algorithm using the Matlab^®^ function fmincon, setting the interior-point algorithm, the continuous-time, and discrete-time constraints.

**Figure 9:**
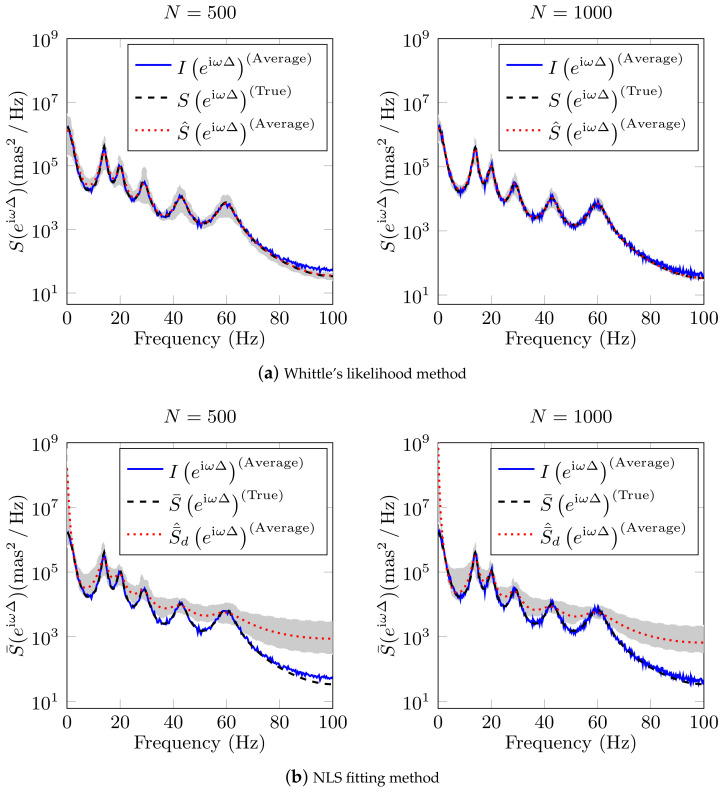
Monte-Carlo simulation results to discrete-time PSD of six disturbance for (**a**) Whittle’s likelihood method and (**b**) NLS fitting method. We use for simulation the values show in Table 1, Δ=5 ms, data length N=500 and N=1000. The solid blue line represents the average of all the periodograms, the dashed black line represents the *true* discrete-time PSD, the dotted red line represents the average of all the estimated PSDs. The gray shaded region represents the area in which all the estimated spectra lie.

Figure 9 shows the estimation results from the MC simulations of the system with six oscillators for a) Whittle’s likelihood method and b) NLS fitting method. Each identification approach yields a different $\left(H_{E}\left(z^{-1}\right)\right)$. In this Figure, the solid blue line represents the average of all the periodograms, the dashed black line represents the *true* discrete-time PSD, the dotted red line represents the average of all the estimated discrete-time PSDs, and the gray shaded region represents the area in which all of the estimated spectra lie. Despite that Whittle’s likelihood is an asymptotic approximation (N→∞) to the traditional likelihood function, in Figure 9 we can observe that the estimation with this approach exhibits good accuracy for small data length, namely N=500. Moreover, we observe that, the greater the *N*, the smaller the gray-shaded region. In contrast, the NLS fitting method does not exhibit an accurate estimation.

Table 3 and Table 4 show the average and standard deviation of all the estimated parameters. The *true* values are presented in Table 1. It is clear that, for the proposed method, the estimates are very accurate, exhibiting a mean value that is almost identical to the *true* values and small standard deviations.

In addition, the dashed-red line frequency response plot shown in Figure 6 corresponds to the estimated model of the disturbances that were obtained with the average of all the estimated parameters using the NLS fitting method in this example.

### 7.2. Performance of MVC in AO System

In order to illustrate the performance of MVC, we consider a mirror gain $M_{0}=1$ and WFS gain $D_{0}=1$. We also select a measurement delay μ=1 and correction delay τ=1, i.e. d=2. The MVC algorithm used in this section is summarised in Algorithm 3.
**Algorithm 3** MVC algorithm1:Compute the disturbance continuous-time model using the Algorithm 2 or other identification method2:Obtain the discrete-time model of disturbance in state-space consider that the sampling period can be different for control3:Compute the spectral factorization4:Compute the coefficients of the finite impulse response filter $F(z^{-1})$ using Equation (59)5:Compute the transfer function $R(z^{-1})$ using Equation (58)6:Compute the controller $K(z^{-1})$ using Equation (61)7:Compute de output variance ${\mathrm{var}}_{y}$ using Equation (66)

On the other hand, Table 5 shows the performance of MVC (*E*), for Δ=5 ms, when using the estimated model that was obtained from both our proposal and the NLS method from [35]. For illustrative purposes, we modified the value of ϑ, from 1 to 0.5 and 1.5, in order to verify how the performance of MVC varies for different values of ϑ. The results show that our method outperforms [35] in every case. In addition, the performance of the MVC does not change significantly when designing the MVC using the estimated model from our proposal for different values of ϑ. The same could not be said about the method in [35]. From these results, we can conclude that our proposal yields more accurate estimates that, in turn, can result in more effective controllers. Note that the performance of MVC for NLS fitting method is the same as that shown in Table 2, i.e., using typical AR(2) model.

**Figure 10:**
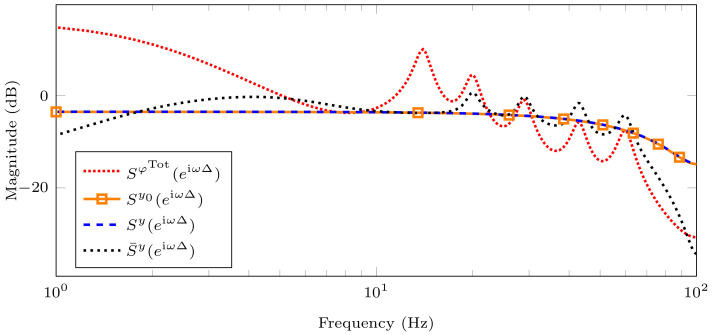
Discrete-time PSD of the disturbance (input signal) and controlled outputs. We use the estimated values that are shown in Table 3 and Table 4, Δ=5 ms, and N=1000. The densely dotted red line represents the discrete-time PSD of disturbance or input signal, the solid orange line with square mark represents discrete-time PSD of the controlled output using the *true* model, the dashed blue line represents discrete-time PSD of the controlled output using the model obtained by Whittle’s likelihood method, the dotted black line represents discrete-time PSD of the controlled output using the model obtained by NLS fitting method.

In Figure 10, we show the discrete-time PSD of the controlled output using the *true* model $S^{y_{0}}\left(e^{i\omega \Delta }\right)$ that is represented by the solid orange line with square marks, the model obtained by Whittle’s likelihood method $\left(S^{y}\left(e^{i\omega \Delta }\right)\right)$ represented by the dashed blue line, and the discrete-time PSD of the controlled output using the AR(2) model that is obtained by the NLS fitting method $\left({\bar{S}}^{y}\left(e^{i\omega \Delta }\right)\right)$ represented by the dotted black line. Note that these controlled outputs are obtained in closed-loop. We also present the discrete-time PSD of the disturbance signal $\left(S^{\varphi^{\mathrm{Tot}}}\left(e^{i\omega \Delta }\right)\right)$ in open-loop represented by the densely dotted red line, corresponding to the input signal. We observe that the controlled output using the proposed modelling and identification methods exhibits better performance than using the NLS method. It is clear that the controlled output obtained with our proposal and the *true* controlled output are almost identical. To see the latter, we compute the normalized mean square error (NMSE), as follows:

(69) $$\mathrm{NMSE}=\frac{\sqrt{\sum_{k=1}^{N}{\left(|S^{y_{0}}\left(e^{i\omega_{k}\Delta }\right)\left|-\right|S^{y}\left(e^{i\omega_{k}\Delta }\right)|\right)}^{2}}}{\sqrt{\sum_{k=1}^{N}{\left(|S^{y_{0}}\left(e^{i\omega_{k}\Delta }\right)|\right)}^{2}}}=0.0067,$$

where |·| denotes the magnitude. We observe that the NMSE value is very small, indicating that both outputs are almost the same and corroborating the benefits of our identification technique on MVC performance.

Note that the controlled output using the estimated AR(2) model and NLS fitting method do not exhibit the disturbance peaks for low frequencies, but, for high frequencies, the disturbances are amplified. This is a consequence of the so-called waterbed effect [48], where the magnitude of the frequency response is reduced in one part of the spectrum, but it is increased in other parts of the spectrum.

Finally we evaluate the effect of the accuracy the estimated model in the control performance when we use a different sampling period, namely Δ=10 ms. The results are the following: E=2.83% for the proposed method and E=18.08% for the NLS. It is clear that the control performance does not exhibit a great variation with respect to the results with Δ=5 ms. Moreover, the new estimates were directly obtained by replacing Δ=0.010 in Equation (46), without the need of running new experiments. In contrast, the approach that is presented in [35] requires carrying out the identification method again with a new sampling period and a new set of samples to obtain the new estimates and then the performance of MVC.

## 8. Discussion and Conclusions

In this paper we addressed the problem of disturbance modelling for minimum variance control in AO systems. We analysed the impact of the accuracy of the disturbance model in the control performance. We showed that the model mismatch (from the parameter estimation) has an impact on the control performance when we used a typical auto-regressive discrete-time disturbance model. We presented a discrete-time model (arising from a continuous-time autoregressive model) for the disturbance that is more accurate at high frequencies.

We obtained a sampled-data state-space model that is more compact and of smaller order than the one that is presented in [30]. We show that the model of the disturbance and the plant can be obtained separately, thus simplifying the design of the controller.

From our simulations we can conclude that Whittle’s likelihood method allows for the identification of continuous-time parameters by directly optimising an explicit function of them. The corresponding cost function is similar to the typical cost function that is obtained from the AR(2) model. However, our proposal yields more accurate estimates whilst maintaining a low computational cost.

We showed that the output of the AO closed-loop system, when using our modelling and estimation proposal, has the best performance, eliminating all of the disturbance peaks for the frequency range of interest. We observed that, when we used the MVC with the correct identification, we improved the performance of controller in 19% with respect to the MVC with the estimated model utilising the classical estimation technique shown in [35].

## Figures and Tables

**Table 1:** The values used for six continuous-time damped oscillators.

$\alpha_{l}\left(\mathrm{Hz}\right)$	2	14	20	29	43	60
$\zeta_{l}$	0.9	0.05	0.05	0.05	0.05	0.05
$\beta_{l}\left(\times 10^{4}\right)$	1.439	3.493	3.734	4.253	5.540	9.711

**Table 2:** Performance of MVC for different disturbance model.

ϑ	0	0.5
E(%)	0	3.85

**Table 3:** Estimated parameters of six disturbance for Whittle’s likelihood method, using N=1000.

	$\hat{\alpha }$ (Hz)	$\hat{\zeta }$	$\hat{\beta }\left(\times 10^{4}\right)$
1	2.143±0.320	0.861±0.152	1.559±0.331
2	14.024±0.194	0.059±0.014	3.775±0.316
3	20.026±0.296	0.055±0.012	3.807±0.287
4	29.010±0.385	0.054±0.010	4.307±0.301
5	43.020±0.473	0.050±0.009	5.503±0.305
6	60.085±0.411	0.050±0.006	9.678±0.416

**Table 4:** Estimated parameters of six disturbance for NLS fitting method, using N=1000.

	α (Hz)	ζ	${\hat{\gamma }}_{d}$
1	0.704±0.424	0.999±0.009	3.665±0.697
2	13.964±0.473	0.077±0.024	14.567±1.595
3	19.930±0.540	0.088±0.097	16.340±7.776
4	28.878±0.813	0.108±0.054	19.363±5.393
5	42.912±0.969	0.142±0.053	24.132±4.714
6	59.794±1.082	0.118±0.033	23.031±3.630

**Table 5:** Performance of MVC (E) for different identifications techniques of disturbance, and different values of ϑ. We use Δ=5 ms, and N=1000.

	$\hat{H}\left(z^{-1}\right)$	
ϑ	**Proposed Method**	**NLS [35]**
0	0	0
0.5	1.56%	3.85%
1	0.870%	16.52%
1.5	2.29%	44.79%

## Data Availability

No new data were created or analysed in this study. Data sharing is not applicable to this article.
